# The laboratory risk indicator for necrotizing fasciitis (LRINEC) scoring: the diagnostic and potential prognostic role

**DOI:** 10.1186/s13049-017-0359-z

**Published:** 2017-03-07

**Authors:** Ayman El-Menyar, Mohammad Asim, Insolvisagan N. Mudali, Ahammed Mekkodathil, Rifat Latifi, Hassan Al-Thani

**Affiliations:** 1 Clinical Medicine, Weill Cornell Medical School, Doha, Qatar; 2 Clinical Research, Trauma Surgery, Hamad General Hospital (HGH), Doha, Qatar; 3 Department of Surgery, Intensive Care Unit, HGH, Doha, Qatar; 4 Department of Surgery, Westchester Health, Valhalla, NY USA; 5 Department of Surgery, trauma Surgery, HGH, Doha, Qatar; 6 Weill Cornell medical college, Clinical Research, Trauma Surgery, Hamad General Hospital, Doha, Qatar

**Keywords:** Necrotizing fasciitis, LRINEC score, SOFA score, Prognosis, Sepsis, Outcomes

## Abstract

**Background:**

Necrotizing fasciitis (NF) is a devastating soft tissue infection associated with potentially poor outcomes. The Laboratory Risk Indicator for Necrotizing Fasciitis (LRINEC) score has been introduced as a diagnostic tool for NF. We aimed to evaluate the prognostic value of LRINEC scoring in NF patients.

**Methods:**

A retrospective analysis was conducted for patients who were admitted with NF between 2000 and 2013. Based on LRINEC points, patients were classified into (Group 1: LRINEC < 6 and Group 2: LRINEC ≥ 6). The 2 groups were analyzed and compared. Primary outcomes were hospital length of stay, septic shock and hospital death.

**Results:**

A total of 294 NF cases were identified with a mean age 50.9 ± 15 years. When compared to Group1, patients in Group 2 were 5 years older (*p* = 0.009), more likely to have diabetes mellitus (61 vs 41%, *p* < 0.001), *Pseudomonas aeruginosa* infection (*p* = 0.004), greater Sequential Organ Failure Assessment (SOFA) score (11.5 ± 3 vs 8 ± 2, *p* = 0.001), and prolonged intensive care (median 7 vs 5 days) and hospital length of stay (22 vs 11 days, *p* = 0.001). Septic shock (37 vs. 15%, *p* = 0.001) and mortality (28.8 vs. 15.0%, *p* = 0.005) were also significantly higher in Group 2 patients. Using Receiver operating curve, cutoff LRINEC point for mortality was 8.5 with area under the curve of 0.64. Pearson correlation analysis showed a significant correlation between LRINEC and SOFA scorings (*r* = 0.51, *p* < 0.002).

**Discussion:**

Early diagnosis, simplified risk stratification and on-time management are vital to achieve better outcomes in patients with NF.

**Conclusions:**

Beside its diagnostic role, LRINEC scoring could predict worse hospital outcomes in patients with NF and simply identify the high-risk patients. However, further prospective studies are needed to support this finding.

## Background

Necrotizing fasciitis (NF) is a rare but rapidly progressive devastating soft tissue necrosis that usually involves fascia and subcutaneous tissues with a significant hospital morbidity and mortality [1–3]. It has been estimated that 13 per million of populations are hospitalized each year for NF, of them 20-30% dies. The mortality rate could reach up to 100% in the absence of the proper and timely diagnosis and treatment [4]. The most common risk factors for NF are diabetes mellitus (DM), immunodeficiency diseases, illicit drug use and malnutrition [1]. This kind of infection can occur with a trivial wound or often without any provocation [5–7]. Early diagnosis, aggressive serial debridement, broad-spectrum antibiotics and multidisciplinary critical care approach are vital to attain favorable outcomes in NF patients [3, 4, 8]. The Laboratory Risk Indicator for NF (LRINEC) is a scoring system driven from six routinely performed laboratory tests and used initially to early distinguishing NF from the other severe soft tissue infections [9]. Multiple studies have assessed the utility of LRINEC for the early diagnosis of NF and found that it can be used for identification and classification of NF patients into different risk categories that subsequently facilitates the appropriate management of hospital resources [10, 11]. However, few studies have observed an association between LRINEC scoring values and outcomes in patients with NF [5, 12–16]. There is always a need to find a simplified bedside, validated, rapid tool to early stratify patients with a potential life-threatening illness. The present study aims to evaluate the role of LRINEC score as a prognostic tool for in-hospital outcomes in patients with NF.

## Methods

A retrospective analysis of prospectively collected data of patients who were admitted to the surgical intensive care unit with a provisional diagnosis of NF regardless of age, sex and ethnicity, was performed. The study was conducted between 2000 and 2013 at Hamad General Hospital (HGH), which is the only tertiary care facility in the state of Qatar. We excluded any cases with incomplete relevant data or inaccurate diagnosis. Collected data included patients’ demographics, clinical presentations, site of infection, type of comorbidities, microbiological and laboratory findings. Primary clinical outcomes were ICU and hospital stay as well as in-hospital mortality.

NF was diagnosed based on clinical and laboratory assessments on arrival and during the hospital stay including clinical criteria by the Center for Disease Control and Prevention and the National Necrotizing Fasciitis Foundation and scoring [1, 2]. When clinical assessment and surgical exploration were equivocal, the final diagnosis of NF in our study was made based on confirmatory histopathologic analysis. Also, a Gram staining at primary debridement is obtained and intraoperative frozen section biopsy is performed whenever there is a suspicion for NF, requiring exploration. Septic shock was defined as sepsis-induced hypotension (i.e., systolic blood pressure less than 90 mmHg (or a fall in systolic blood pressure of > 40 mmHg) persisting despite adequate fluid resuscitation [12]. LRINEC was calculated at presentation using laboratory results of six variables C-reactive protein (CRP), white blood cell count, hemoglobin, sodium level, creatinine and glucose (Table 1) [9]. For this study analysis, we included only patients who fulfilled the required laboratory findings to calculate LRINEC score. Patients were stratified according to the LRINEC scoring points into two groups; score <6 (Group 1) and ≥6 (Group 2) to study different characteristics and outcomes. SOFA (Sequential Organ Failure Assessment) score was calculated using parameters such as the ratio of partial pressure arterial oxygen and fraction of inspired oxygen (PaO2/FiO2), platelets count, bilirubin level, Glasgow coma score, Mean Arterial Pressure (MAP), use of vasopressors, creatinine level and urine output [17]. NF has been classified into different groups (I-IV) based on microbiological cultures (Table 1) [18].

**Table 1:** Study definitions

Laboratory Risk Indicator For Necrotizing Fasciitis	
Variable (units)	Score points
C-Reactive Protein (CRP) (mg/L)	
<150 >150	0 4
White blood cell count (per mm^3^)	
<15 15-25 >25	0 1 2
Hemoglobin (g/dl)	
>13.5 11-13.5 <11	0 1 2
Serum Sodium (mmol/L)	
≥135 <135	0 2
Serum Creatinine (mg/dl)	
≤1.6 >1.6	0 2
Serum Glucose (mg/dl)	
≤180 >180	0 1
Types of NF based on microorganisms	
Type I	NF comprised of synergistic polymicrobial infection
Type II	NF is caused by monomicrobial gram positive organisms
Type III	NF involves gram negative monobacteria usually marine-related organisms
Type IV	NF is caused by fungal infection

The standard dressing management: After the initial debridement, the surgeon would use antiseptic soaked gauze dressing to absorb the expected post-debridement ooze. Following an adequate debridement, wet to dry dressings started 2–3 times a day to achieve gentle debridement of residual sloughs and debris otherwise difficult to clear surgically. Once clean wound is achieved, a vacuum assisted closure (VAC) could be used to enhance healing and formation of healthy granulation tissues and reduce wound surface area. The plan for secondary closure is often discussed with reconstructive plastic surgeons at early stage.

### Statistical analysis

Data were presented as proportions, median (range) or mean (± standard deviation), as appropriate. Baseline demographic characteristics, medical history, SOFA score on admission, initial procalcitonin (PCT) levels and outcomes were compared between the two groups according to LRINEC values on admission. Analyses were conducted using Student *t* test or ANOVA test for continuous variables and Pearson chi-square (*χ* ^2^) test for categorical variables, whenever applicable. Non-parametric Mann-Whitney test was used for skewed variables. A 2-tailed *p* < 0.05 was considered significant. Receiver–operator characteristic curves were plotted to identify LRINEC cut-off point for predicting septic shock and mortality. Area under the curve (AUC) was used to compare the discriminatory power of the scoring system or other clinical variables of interest, with an AUC 1.0 considered perfect discrimination and 0.5 considered equal to chance [19]. The correlation between LRINEC and SOFA values was performed using Pearson correlation that was considered significant at the 0.01 level (2-tailed). We also sub-analyzed data to look for outcomes in a subset of patients with no records of LRINEC scores to avoid selection bias. Data analysis was carried out using the Statistical Package for Social Sciences version 18 (SPSS Inc, Chicago, Illinois).

## Results

**Fig. 1:**
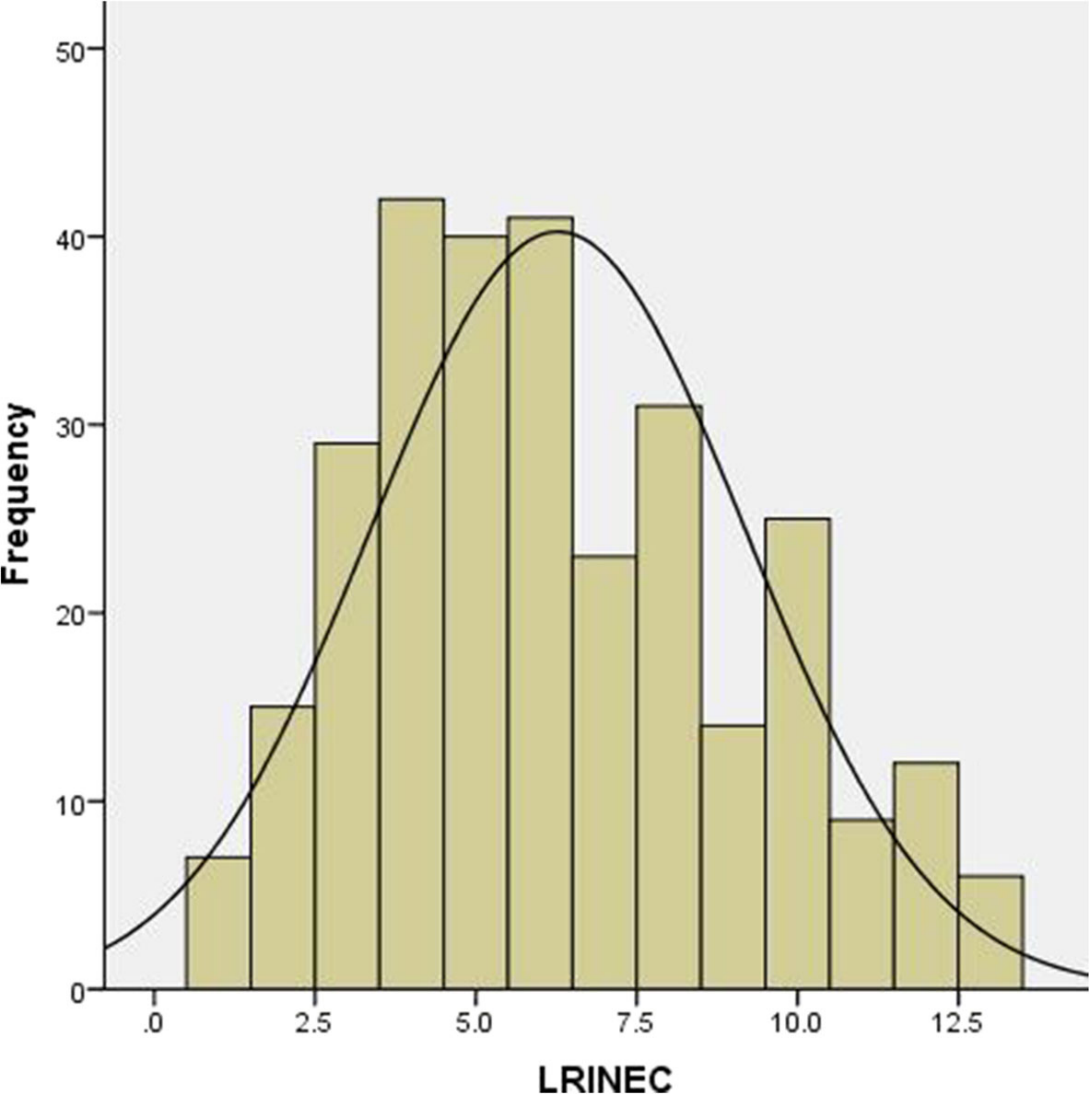
Frequency of LRINEC scoring [n=294, mean±SD (6.3±2.9)]

During the study period, 331 NF cases were hospitalized and LRINEC score was successfully calculated in 294 cases. One hundred and thirty three (45%) of these patients were included in Group 1 and 161 (55%) in Group 2. Mean LRINEC score on admission was 6.28 ± 2.9. Figure 1 shows the normal distribution of mean LRINEC scores. Males were predominant in the overall patient cohort (217; 73.8%); and the proportion of patients by gender was comparable between the 2 groups (*p* = 0.82). Table 2 summarizes patients’ characteristics in the 2 study groups. The mean age of patients was 50.9 ± 15 years. When compared to Group 1, patients in Group 2 were five years older (mean age 53.1 ± 15.7vs. 48.4 ± 14.9 years, *p* = 0.009); more likely to have comorbidities such as DM (61.4 vs 41.5%, *p* < 0.001); hypertension (46.8 vs. 21.5%, *p* = 0.001); and renal disease (20.3 vs. 10.0%, *p* = 0.02). Group 2 also had higher proportion of pseudomonas aeruginosa infections than those in Group 1 (11.7 vs. 2.4%, *p* = 0.004). LRINEC values were higher in NF type IV, however, no significant statistical differences between the two groups were seen in terms of the other microorganisms, or site of infections (Table 3).

**Table 2:** Demographics, comorbidities, region of infection and outcomes in patients with NF

	LRINEC <6 (45%)	LRINEC ≥6 (55%)	P
Age, years (mean ± SD)	48 ± 15	53 ± 16	0.009
Males (%)	74	73	0.82
Diabetes Mellitus (%)	41.5	61.4	0.001
Kidney disease %	10	20	0.02
Hypertension%	28 (21.5)	74 (46.8)	0.001
Site of infection %			
Lower limbs	49	55	0.32
Perineum & genitalia	35.3	34.8	0.92
Abdominal and groin	12.0	8.7	0.34
Chest & breast	3.0	2.5	0.78
Face & neck	6.0	7.5	0.62
Number of debridement	2.14 ± 1.5	2.09 ± 1.3	0.81
Number of antibiotics used^a^ (%)			
≤2	81 (78.6)	83 (56.8)	0.001 for all
>2	22 (21.4)	63 (43.2)	
Hospital LOS; days	11 (2–115)	22 (2–129)	0.001
intensive care LOS; days	5 (2–34)	7 (1–75)	0.01
Septic shock (%)	15	37	0.001
Mortality (%)	15	28.8	0.005

^a^Frequently used antibiotics are Tazocin, Clindamycin, Meropenem and Agumentin

**Table 3:** Laboratory results

	LRINEC <6 (45%)	LRINEC ≥6 (55%)	*P*-value
Streptococcus (%)	51(40.8)	45 (33.1)	0.19
Staphylococcus (%)	47(37.6)	49 (35.8)	0.76
Bacteroides (%)	30 (24.0)	26 (18.9)	0.32
Escherichia coli (%)	15 (12)	16 (11.6)	0.92
Pseudomonas (%)	3 (2.4)	16 (11.7)	0.004
Proteus mirabilis (%)	0 (0)	5(3.6)	0.06
Gram positive (%)	94 (82.5)	99 (76.2)	0.09
Gram negative (%)	48 (42.1)	71 (54.6)	0.08
Causative bacteria (%)			
Type I (polybacterial) (%)	36 (31.6)	38 (29.2)	0.06 for all
Type II (Monobacterial) (%)	70 (61.4)	70 (53.8)	
Type III (Murine bacteria) (%)	0 (0)	0 (0)	
Type IV (Fungal) (%)	8 (7.0)	22 (16.9)	
C-Reactive protein level, mean±SD	119 ± 82	249 ± 111	0.001
Initial Procalcitonin level, median (range)	0.85(0.09-182)	8.1(0.07-303)	0.127
SOFA score			
Mean	8.7 ± 2.4	11.6 ± 3.3	0.001
Median	8 (2-19)	11 (4-21)	
LRINEC score			
Mean	3.7 ± 1.1	8.4 ± 2.1	0.001
Median	4 (1-5)	8 (6-13)	

The confirmatory histopathology was found in 192 patients (65%), whereas there were no histopathology data available for the remaining patients. However, the mean LRINEC scores were almost similar in patients who had histopathology and those who had no histopathology reports (6.1 vs 6.4 points).

Initial PCT levels [median 8 (0.07-303) vs. 0.8 (0.09-182)] and SOFA score on admission (11.6 ± 3.3 *vs* 8.7 ± 2.4, *p* = 0.001) were greater in Group 2 patients than those in Group 1. Pearson correlation for LRINEC and SOFA scores showed moderate correlation: *r* = 0.51, *p* < 0.001). The intensive care duration (median 7 (1-75) *vs* 5 (2-34) days and hospital length of stay [22 (2-129) *vs* 11 (2-115) days, *p* = 0.001] were greater in the Group 2 patients. Septic shock and mortality rates were 26 and 22% respectively in the cohort. The proportion of septic shock (37.1% vs. 15.2%, *p* = 0.001) and mortality (28.8 vs. 15.1, *p* = 0.005) were higher in Group 2 patients than Group 1 (Table 2). Also, significantly a higher proportion of patients in Group-2 required administration of more than two types of antibiotics (43.2% vs. 21.4%; *p* = 0.001). Age-adjusted LRINEC values as predictors of septic shock and mortality are shown in Table 4.

**Table 4:** Predictors of hospital outcomes in NF

	Risk of Mortality		
	Odds ratio	95% CI	*P* value
LRINEC score	1.20	1.09–1.29	0.01
Age	1.07	1.04–1.09	0.001
Risk of Septic Shock			
LRINEC score	1.30	1.15–1.41	0.001
Age	1.02	1.001–1.041	0.042

### Temporal relationship between mortality and LRINEC scores

**Fig. 2:**
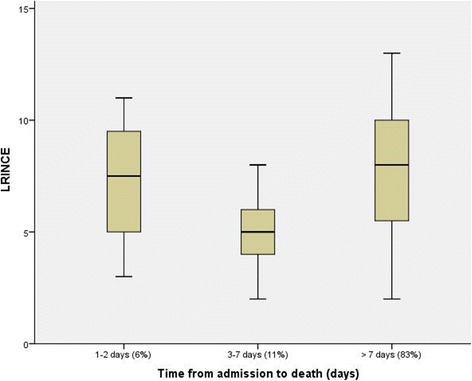
A Boxplot chart of the time to death in NF patients based on the LRINEC scoring

Time to death in NF patients based on the LRINEC scoring is shown in Fig. 2. Although this Boxplot chart shows a U-shape death pattern, the majority of deaths occurred after the first week of admission (83%). The mean LRINEC scores were higher for those who died after the first week (late) and within the first 2 days post-admission(early) than who died between the 3^rd^ and 7^th^ day post-admission (7.7 ± 3.0, 7.2 ± 3.3 and 2.1 ± 1.0, respectively), *p* = 0.03.

**Fig. 3:**
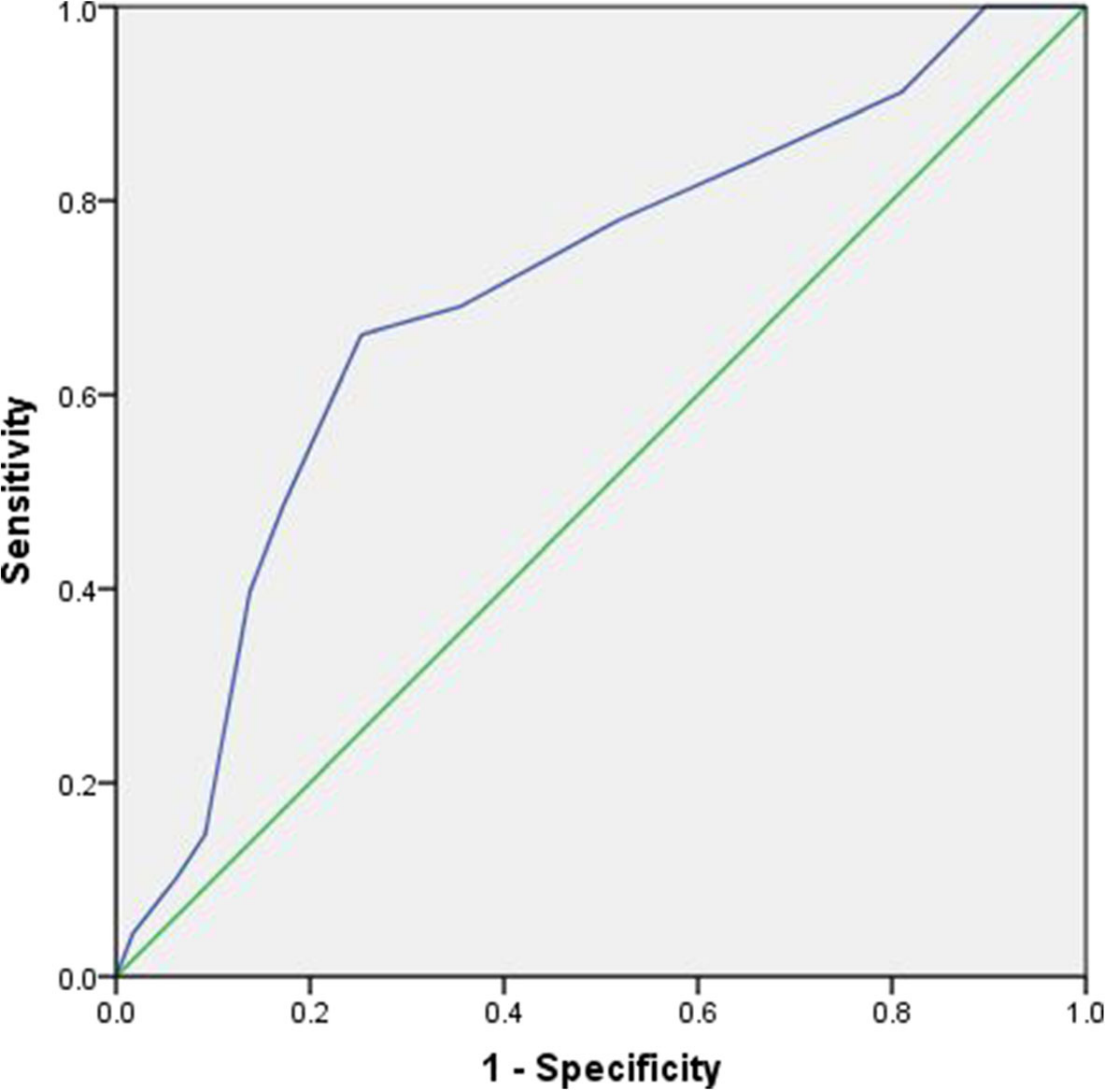
ROC curve for LRINEC scoring points in the prediction of septic shock: Area under the curve 0.70; 95% confidence interval 0.63-0.78, p<0.001. LRINEC scoring cut-off value 5 with 82% sensitivity and 72% specificity

**Fig. 4:**
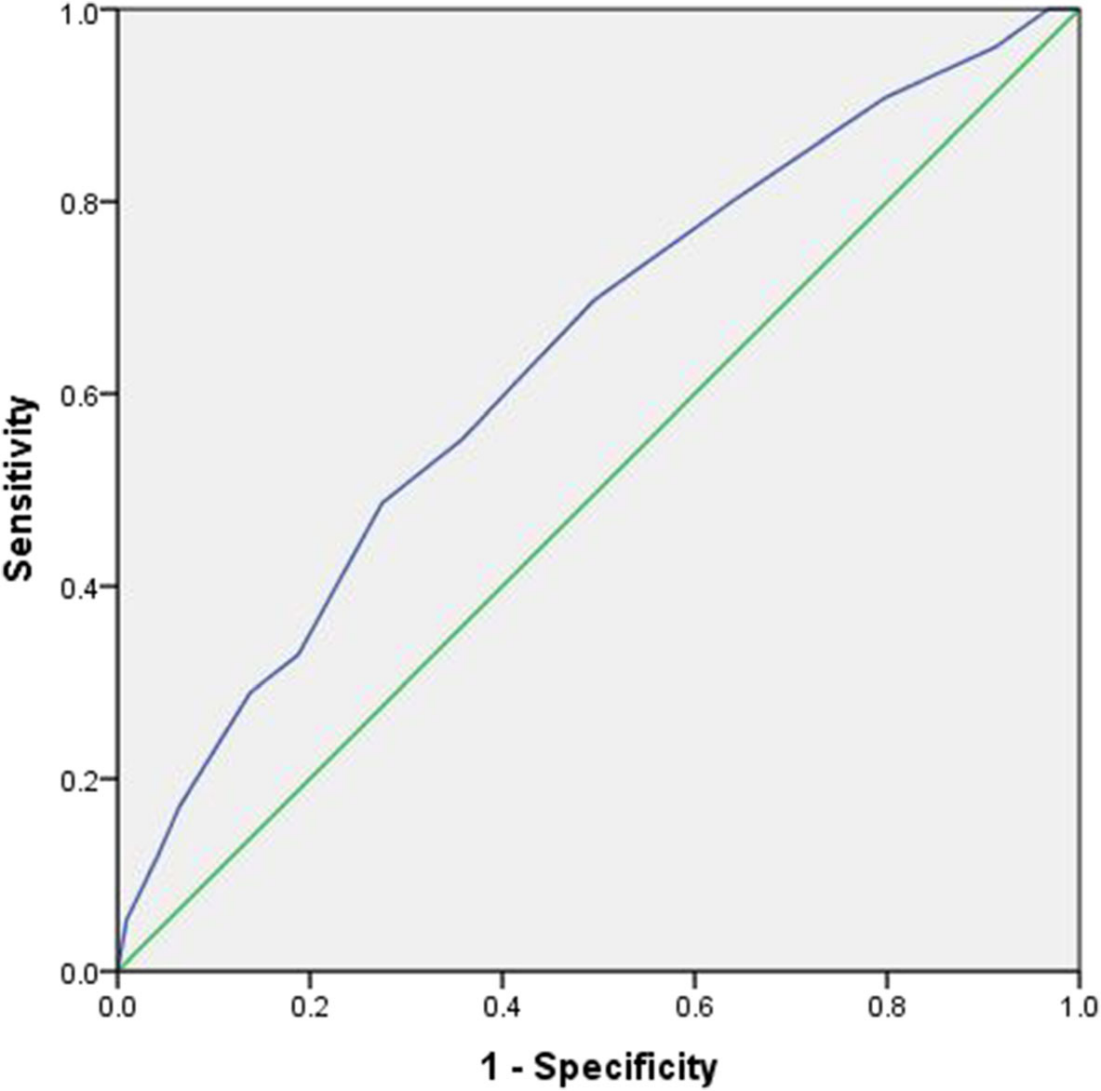
ROC curve for LRINEC scoring points in the prediction of mortality: Area under the curve 0.64; 95% confidence interval 0.57-0.71, p<0.001. LRINEC scoring cut- off value 8.5 with 81% sensitivity and 36% specificity

The cut-off point of LRINEC scoring for predicting septic shock was 5 points (sensitivity 82% and specificity 38%) whereas the cut-off point for predicting mortality was 8 points (sensitivity 81% and specificity 36%). Figures 3 and 4 demonstrate the ROC curves for LRINEC scoring points in the prediction of septic shock and mortality.

Table 5 shows a sub-analysis including patients in whom LRINEC scoring was not calculated or documented (*n* = 37). Patients without LRINEC scoring had the lowest SOFA score in comparison to the other LRINEC groups. Patients in Group 2 had higher rates of septic shock and mortality than Group 1 as well as the non-documented LRINEC group.

**Table 5:** Outcomes based on LRINEC status

	Not available	LRINEC <6(group1)	LRINEC ≥6(group2)	P
Patients number	37	133	161	
SOFA score; mean ± SD	7.6 ± 1.1	8.7 ± 2.3	11.5 ± 3.3	0.001
Septic shock %	25.8	15.2	37	0.001
Mortality%	18.9	15	28.6	0.05

## Discussion

In patients with NF, early diagnosis, simplified risk stratification and on-time, appropriate surgical debridement are crucial to achieve better outcomes [9, 20]. There are few studies which indicate that LRINEC scoring is a useful diagnostic tool with a potential prognostic value. Although most of these studies were not primarily aiming at the evaluation of the prognostic role of LRINEC score, it revealed poor outcomes in NF patients with high LRINEC scores [5, 12, 14–16]. Moreover, most of these studies were characterized by being of small sample size (15–209 cases). The present study aims primarily to assess the prognostic value of LRINEC in 294 NF patients. In our study, male to female ratio was relatively higher (2.8) in comparison to prior studies (1.7 to 2.3), however, the average age of patients (51 years) was comparable. Increased age and other comorbidities such as DM, hypertension, obesity, peripheral vascular disease and renal impairment were found to be associated with unfavorable outcomes including limb loss and mortality in NF patients [1, 14, 16, 21, 22]. Notably, DM is the most common co-morbidity reported in NF patients; with a prevalence of up to 2 out of 3 cases [5, 13–16]. In our series, DM, hypertension and kidney diseases were the most frequent comorbidities in patients with LRINEC score ≥6.

Similar to the previous study of Glass et al study; streptococcus was the most frequently identified pathogen in the present cohort [23]. However, there was no difference between the two LRINEC study groups in terms of the other causative organisms except for pseudomonas species. Patients with higher LRINEC scores were more likely affected by pseudomonas. Colak et al demonstrated that pseudomonas aeruginosa infection was significantly higher in the non-surviving group when compared to the survivors [15].

The biological variables used to calculate LRINEC scoring are found to correlate individually with the diagnosis of NF in some studies. For example, a prospective observational study showed that WBC count >15,400/ microL or serum sodium <135 mEq/L significantly increased likelihood of NF diagnosis [20]. Wong et al found the cut-off value for the LRINEC score to detect early cases of NF as 6 points with a positive predictive value of 92% and negative predictive value of 96% [9].

Chao et al [10] demonstrated that in 125 patients diagnosed with NF, LRINEC score of ≥2 had a sensitivity of 71%, a specificity of 83%, with an 12-fold increased risk for the presence of NF due to Vibiro vulnificus (*n* = 72). Holland et al also in a small retrospective study (*n* = 28), evaluated the effectiveness of LRINEC cut-off score ≥ six [24]. This cut-off score showed a sensitivity of 80%, specificity of 67%, a positive predictive value of 57% and a negative predictive value of 86% in distinguishing patients with proven NF from those with severe soft tissue infections [24]. However, the negative predictive value of LRINEC was questioned recently in a cohort of 24 patients with histologically confirmed NF [23].

There are few case-reports assessed the applicability of LRINEC score in the diagnosis of NF. Wilson and Schneir reported a case of NF confirmed at surgery where LRINEC score was zero [25]. Whereas, Kulkarni et al reported a sharp increase in LRINEC score (from 7–11) in one case on the 5^th^ day of admission due to an increase in CRP level [2]. Despite of the early diagnosis and aggressive intervention, that patient was not survived due to immunocompromised condition and aggressive polybacterial infection.

The prognostic potential of LRINEC score has been reported in few studies as shown in Table 6 [5, 12, 14–16]. Su et al studied the LRINEC cut-off value associated with poor outcomes in 209 NF patients and demonstrated higher mortality and amputation rates in patients based on LRINEC scores [5]. The rates of early diagnosis (64 vs 70%), early operation (71 vs 70%) and time for operation (30 ± 51 vs 27.5 ± 51 min) were comparable between the 2 LRINEC groups. The overall mortality and amputation rates were 16 and 26%, respectively. Whereas, the rates of mortality (21% vs. 11%) and amputation (36% vs. 17%) in patients with LRINEC score ≥6 were higher than those who had LRINEC < 6 [5].

**Table 6:** Summary of studies on the prognostic role of LRINEC in NF patients

Study	Country	Design	Results
Su et al. [5]	Taiwan	Retrospective study (2002–2005) *N* = 209	Patients with a LRINEC score of ≥6 have a higher rate of both mortality and amputation.
Corbin et al. [12]	France	Prospective study *N* = 50	The rate of complications was higher for patients with a LRINEC score > 6 than for patients with a score < 6.
Swain et al. [14]	UK	Retrospective study (2006–2011) *N* = 15	Overall mortality was 3 out of 15 patients. The median LRINEC score in all deaths was 9.0 (range: 6–12).
Bozkurt et al. [16]	Turkey	Retrospective study (2008–2013) *N* = 33	Patients with higher LRINEC scores were more likely to require mechanical ventilation and longer hospitalization times and were more likely to die
COLAK et al. [15]	Turkey	Retrospective study (2008–2013 *N* = 25	The mean number of debridements and LRINEC score were higher in the non-surviving group (*p* = 0.003 and *p* = 0.003, respectively).
El-Menyar et al. 2017	Qatar	Retrospective study 2000–2013 *N* = 294	LRINEC ≥6 had greater SOFA score (11.5 ± 3 vs 8 ± 2) septic shock (37% vs 15%), prolonged hospital length of stay and deaths (*p* < 0.001 for all)

Corbin et al prospectively studied the prognostic value of LRINEC score in 50 patients [12]. The rate of complications (septic shock, transfer to intensive care and mortality) in NF patients with LRINEC ≥6 was higher when compared to the patients with score <6. Bozkurt et al evaluated this capability of LRINEC in predicting the morbidity and mortality in patients with Fournier's gangrene (*n* = 33) [16]. In that study, LRINEC score effectively predicted the requirement of mechanical ventilation and mortality. Colak et al also found that high LRINEC scoring might predict the requirement of debridement and mortality in NF patients (*n* = 25) [15]. Our study is in consistency with these reports that showed significantly higher mortality and septic shock rates in patients with LRINEC score ≥ 6. In addition, the duration of stay in ICU and hospital were significantly longer among patients with higher scores. Also, the present study showed that although the number of debridement was comparable in the 2 LRINEC groups, the number of antibiotics used was higher in patients with greater LRINEC scoring. Our analysis showed that the number of NF- related in-hospital deaths was increasing over the time. Notably, the mortality and LRINEC scores had a U-shape relationship. The mean scores were significantly greater in those who died after the first week of admission in contrast to its lower value in those who died between the 3^rd^ and 7^th^ day.

Of note, the other severity scoring systems like APACHE and SOFA were scarcely tested as predictors of outcome in patients with NF, and if so, this was as a surrogate outcome [26–30]. Patients with SOFA scores of more than >8 were found to deserve urgent admission to the surgical intensive care and also to be associated with poor outcome in patients with NF [26, 30].

In our study, SOFA was significantly higher in patients with LRINEC ≥6 (mean SOFA 11.6 ± 3.3) when compared to patients with LRINEC <6 (mean SOFA 8.7 ± 2.4). Our analysis demonstrated that Pearson correlation for LRINEC and SOFA scores showed a moderate correlation (*p* < 0.001). LRINEC cut-off value for predicting hospital mortality was 8 points in our study. Notably, Swain et al reported 20% mortality in their NF patient population, and the median LRINEC score of all patients who died was nine [14]. However, the low specificity for LRINEC cut-off for predicting mortality (cut off value = 8, sensitivity 81% and specificity 36%) as well as septic shock (cut off value = 5, sensitivity 82% and specificity 38%) are of concern and need further prospective evaluation. Furthermore, research work is still needed to determine the utility of LRINEC scoring and to validate its prognostic role in the hospital outcomes in patients with NF.

**Fig. 5:**
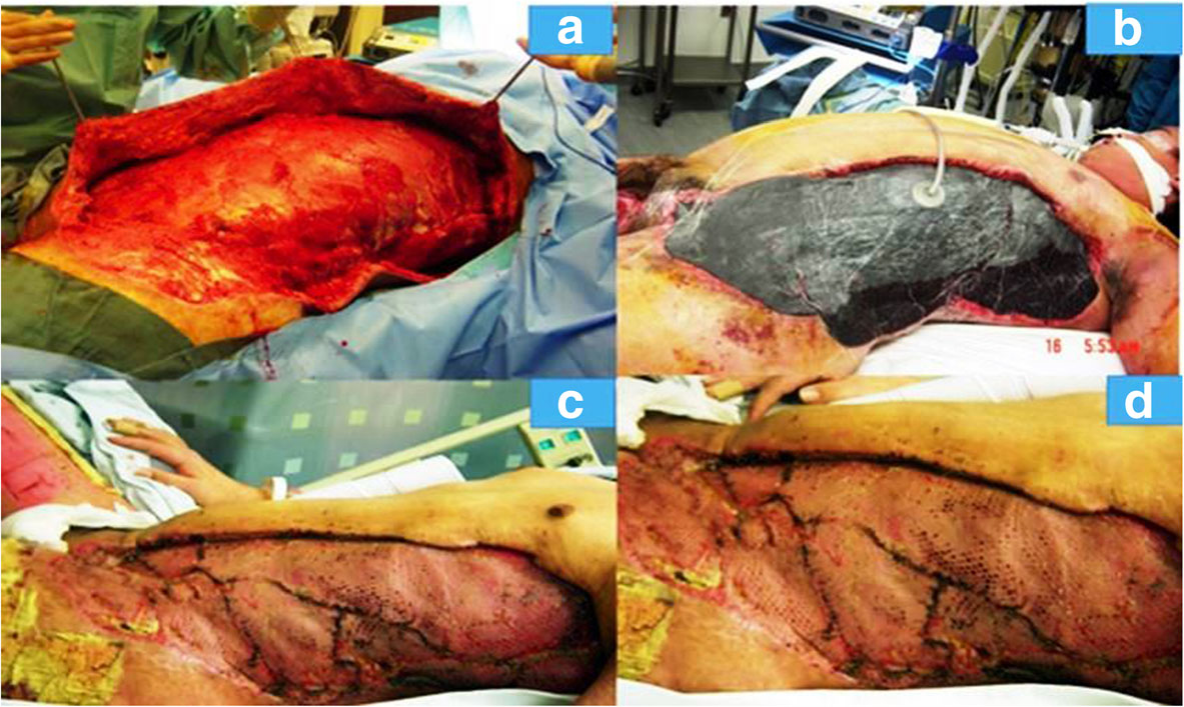
Patient with NF of left chest wall extending from left axilla to left groin and scrotum. **a**) Post-surgical debridement of the chest and abdominal wall; **b**) Wound VAC placement on the same patient; **c** & **d**: Two consecutive images of reconstruction of the same wound with skin graft

Finally, one of the most important elements of NF is the surgical management, which needs to commence as soon as possible [31] in terms of consist of aggressive and wide debridement. More often than note, these patients undergo a number of debridements and meticulous wound management including the nowadays VAC and skin graft reconstruction (Fig. 5).

### Limitations

The retrospective nature of this study is a limitation to fill the gaps and to generalize the results. There were 37 cases (out of the 331 NF patients) in which LRINEC scoring was not available in our database, however, we analyzed their data to avoid selection bias. The mortality rate was 18.9% in this subgroup (no LRINEC) in comparison to 28.6% in high LRINEC group and 15% in the low LRINEC scoring group. This finding supports the need to measure LRINEC in all NF cases. The reason behind missing LRINEC in some cases was not clearly identified. According to database registry, confirmatory hisopathology results were not available for one third of the cases. Further, prospective studies are needed to validate the scoring system for this purpose. Determination of the time interval between the diagnosis and treatment (medical and surgical) could possibly influence the outcome in the NF patients as there is evidence that delayed first debridement is often associated with poor outcomes [20], while operating early has reduced hospital and ICU length of stay [31]. Also, the effect of possible prior antibiotic treatment from other facilities was not addressed or reported. The percent total body surface as well as the exact time intervals for debridement and dressings was not studied. Therefore, we are currently using this observation as an audit to fix these gaps.

## Conclusions

In patients presented with NF, LRINEC scoring, in addition to its diagnostic role, could be used for risk stratification and prognosis. Further prospective studies are needed to support and validate our findings.

## Abbreviations

APACHE: Acute Physiology and Chronic Health Evaluation
LRINEC: The Laboratory Risk Indicator for Necrotizing Fasciitis
NF: Necrotizing fasciitis
SOFA: Sequential Organ Failure Assessment
WBC: white blood cell count
